# Pure edge-contact devices on single-layer-CVD-graphene integrated into a single chip

**DOI:** 10.1038/s41598-023-37487-1

**Published:** 2023-06-30

**Authors:** Saraswati Behera

**Affiliations:** Department of Microtechnology and Nanoscience (MC2), Chalmers University of Technology, Gothenburg, Sweden

**Keywords:** Synthesis of graphene, Design, synthesis and processing, Electronic properties and devices

## Abstract

We present a simple and cost-effective fabrication technique for on-chip integration of pure edge contact two-terminal (2T) and Graphene field effect transistor (GFET) devices with low contact resistance and nonlinear characteristics based on single-layer chemical-vapor-deposited (CVD) graphene. We use a smart print-based mask projection technique with a 10X magnification objective lens for maskless lithography followed by thermal evaporation of the contact material Cr-Pd-Au through three different angles (90° and ± 45°) using a customized inclined-angle sample-holder to control the angle during normal incidence evaporation for edge-contact to graphene. Our fabrication technique, graphene quality, and contact geometry enable pure metal contact to 2D single-layer graphene allowing electron transport through the 1D atomic edge of graphene. Our devices show some signatures of edge contact to graphene in terms of very low contact resistance of 23.5 Ω, the sheet resistance of 11.5 Ω, and sharp nonlinear voltage-current characteristics (VCC) which are highly sensitive to the bias voltage. This study may find application in future graphene-integrated chip-scale passive or active low-power electronic devices.

## Introduction

Graphene is considered a suitable 2D material for optical, electronic, and optoelectronic applications due to its superior physical properties such as lightweight, charge carrier mobility, large surface area, high electrical and thermal conductivity, zero and tunable bandgap and optical transparency^[1–5]^. Many researchers and industry are hopeful about the integration of graphene into silicon technology towards technological revolutions in active and passive micro and nanoelectronic devices. However, the metal-to-graphene contact resistance is a key parameter when considering the CMOS compatibility of graphene^6,7^. The contact resistance’s nature depends on the physical interaction between the metal and the atomically thin graphene for the injection of charge carriers into the channel region^7,8^. An electrostatic field is created when the metal comes in contact with the graphene atoms and prohibits the further transfer of carriers in the graphene channel. The more, the electrostatic field at the junction, the higher the applied field required to transport carriers inside the channel and vice-versa. Therefore, it is important to reduce the electrostatic field near the metal-graphene junction either through physical changes to the geometry at the metal-graphene interface or through chemical treatments to graphene or contact metals^8–10^. Contact resistance also plays a role to tune the charge neutrality point in multi-layer graphene for several applications^11^.

There are wide studies reported in this direction for the reduction of metal-graphene contact resistance through the active region or contact geometry^12–14^, choice of contact material^8,9,15^, doping to graphene^10^, contact-pad patterning^8,10^, chemical and thermal treatments to graphene^9,15^, UV treatments to graphene^16^ and edge contact to graphene^9,10,14,17–20^. So far, edge contact to graphene is the most successful technique to improve the M-G contact resistance. It can be done either through chemically doping the graphene, UV exposure to modify the chemical bonds in the graphene, or through physical contact geometry to graphene, which also indirectly dopes the graphene. Moreover, from a device robustness and performance reproducibility point of view, a contact geometry-based edge contact is comparatively more effective. Recently, Park et al.^10^ have presented an edge-top contact design including patterned TLMs below the graphene contact electrode that shows reduced contact resistances which can also be extremely reduced through a combination of edge contact and n-type doping to graphene. A similar study is carried out by Song et al.^18^ by patterning the contact electrode to graphene for improved edge contact. However, these multi-step techniques allow chemical doping to the graphene edge and are partially edge-contacted.

There are limited studies reported on pure edge contact devices to CVD graphene for improvement of metal–graphene contact resistance. A pure 1D edge contact device is presented by Hemmetter et al.^17^ through asymmetric Ni–Au and Ti–Au contacts to graphene, which is highly nonlinear, whereas it shows a total resistance of 74 KΩ between contacts to the graphene. Wang et al.^13^ have presented 1D edge contact to exfoliated 2D graphene sandwiched between two layers of boron nitride (BN) and show low contact resistances of the order of 100 Ω at low temperatures. Further, most of the devices with low contact resistances are based either on exfoliated graphene or epitaxial graphene^13,22^. Here, in our study, (1) we can grow and transfer single-layer graphene using CVD, (2) integrate it to two-terminal devices using a cost-effective and simple fabrication approach as described below. (3) As the signature of a pure 1D edge-contact geometry, we have achieved much-improved contact resistances at room temperature compared to those presented in the literature^13,17,18,21^, nonlinear total resistance variation concerning graphene channel length and a voltage-sensitive nonlinear voltage-current characteristic reflecting the nature of charge carrier transport from metal contacts through 1D atomic-edges of single layer-graphene. (4) Next, we have also verified the nonlinear resistance variation in our devices through the fabrication of edge-contacted TLM sets with variable graphene channel length and width. There are wide studies reported on the transport properties and VCC of graphene using field effect devices under several conditions such as channel length scalability, doping to contact electrode, and top gate interface-trap engineering through top-gate, etc.^23–25^. Therefore, (5) to understand the edge-contact effects on transport properties and the Dirac point of graphene, and the edge-enhanced nonlinearities in VCC, we have included a separate study on the edge-contact graphene field effect transistor (GFET) device. Initially, we fabricated an edge-contacted 2 T device with a graphene channel width of 200 µm and a channel length of 30 µm using the same technique, and then we added a top gate insulated by 120 nm of Parylene N.

Our devices show a contact resistance of 23.5 Ω leading to 0.117 Ω per µm (4.7 K Ω µm) and a contact resistivity of 1.4 Ω µm^2^ through Cr–Pd–Au contact to pure plasma etched 1D edge-of the graphene. These devices may find application in future graphene-integrated low-power electronic devices and fast-charging electrodes.

## Results and discussion

**Figure 1:**
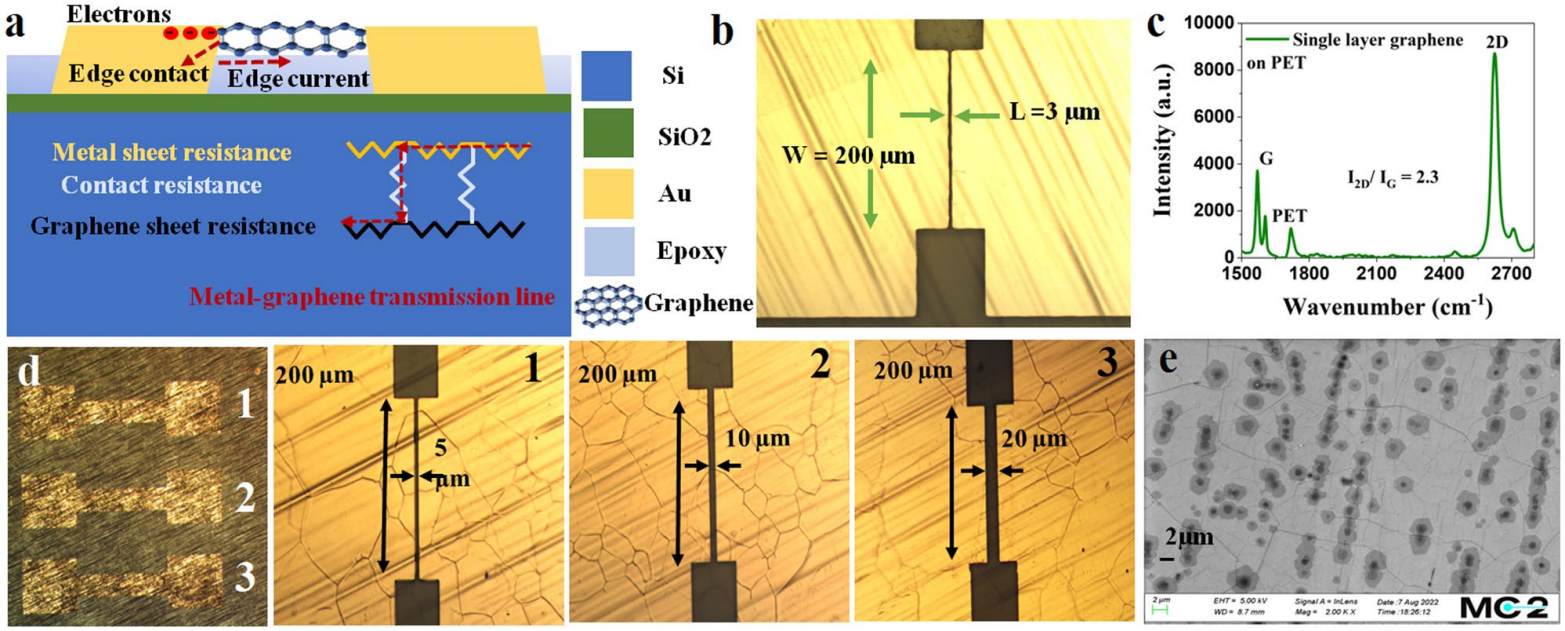
(**a**) Schematic of the edge-contact MG device, (**b**) optical microscope image of the device showing 3 µm graphene channel length and 200 µm graphene width, (**c**) Raman signal of the single layer graphene on polyethylene-terephthalate (PET) that is transferred to the device, (**d**) optical microscope image of the devices (1–3) with variable parameters realized on a single chip, and (**e**) SEM image of the graphene transferred to PET.

The one-dimensional schematic of the Cr-Pd-Au edge contact device is presented in Fig. 1a, where the Cr–Pd–Au edge is in contact with the 1D edge of the graphene atoms. This is the only contact for charge carrier transport in graphene; we observe that these devices show lesser contact resistance (< 40 Ω between two electrodes) than our previously fabricated top contact devices with the same contact geometry showing > 1 K Ω between two electrodes. The inset in Fig. 1a shows the metal-graphene transmission line which is similar to the model presented in^26^. The microscope image of the device with a graphene channel length of 3 µm, and channel width of 200 µm is shown in Fig. 1b. The Raman signal of the single-layer graphene used in our devices is transferred to PET as shown in Fig. 1c. To show our fabrication's robustness and scalability, we repeated this fabrication for three devices with variable channel lengths integrated over a 7 × 7 mm chip as shown in Fig. 1d. The microscope images of three devices in Fig. 1d reveal channel lengths of 5, 10, and 20 µm. The SEM image of the graphene on PET is shown in Fig. 1e, where, patches show the multi-layer regions and non-patch regions present the single-layer graphene.

**Figure 2:**
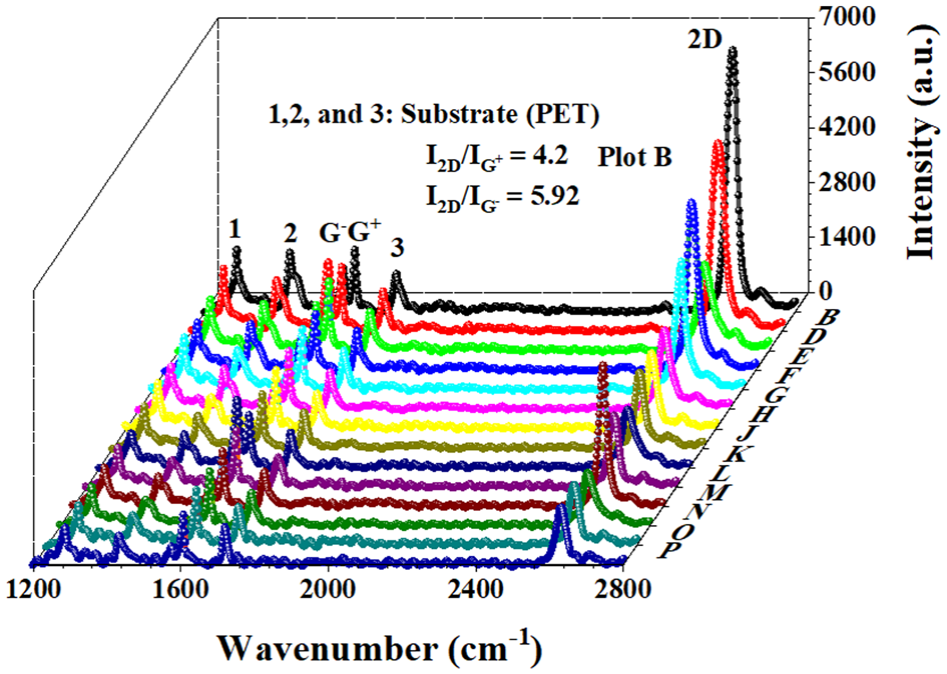
Raman map of the sample over an area of 10 µm^2^ showing signatures of high-quality single-layer graphene.

A Raman map over an area of 10 µm^2^ from the patch region towards the non-patch region is presented in Fig. 2. The Raman map presents the signature of single-layer graphene with a higher 2D peak compared to the G Peak with a maximum I_2D_/ I_G_^+^ of 4.2 and I_2D_/ I_G_^−^ of 5.92. Due to the stretching of the Graphene plane during PET transfer, a splitted G peak appears with G^−^ at 1574 cm^−1^ and a blue-shifted G^+^ peak at 1608 cm^−1^. We have a negligible D peak at 1345 cm^−1^, and the 2D peak is at 2630 cm^−1^. Rests of the peaks are due to the PET substrate. A 2D plot of the Raman map is presented in the supplementary material (Fig. S1). The transferred graphene on PET shows stable mobility of 6480 cm^2^ V^−1^ S^−1^ which also drastically increases to 30,000 cm^2^ V^−1^ S^−1^ upon heating at 60 °C for a few minutes.

**Figure 3:**
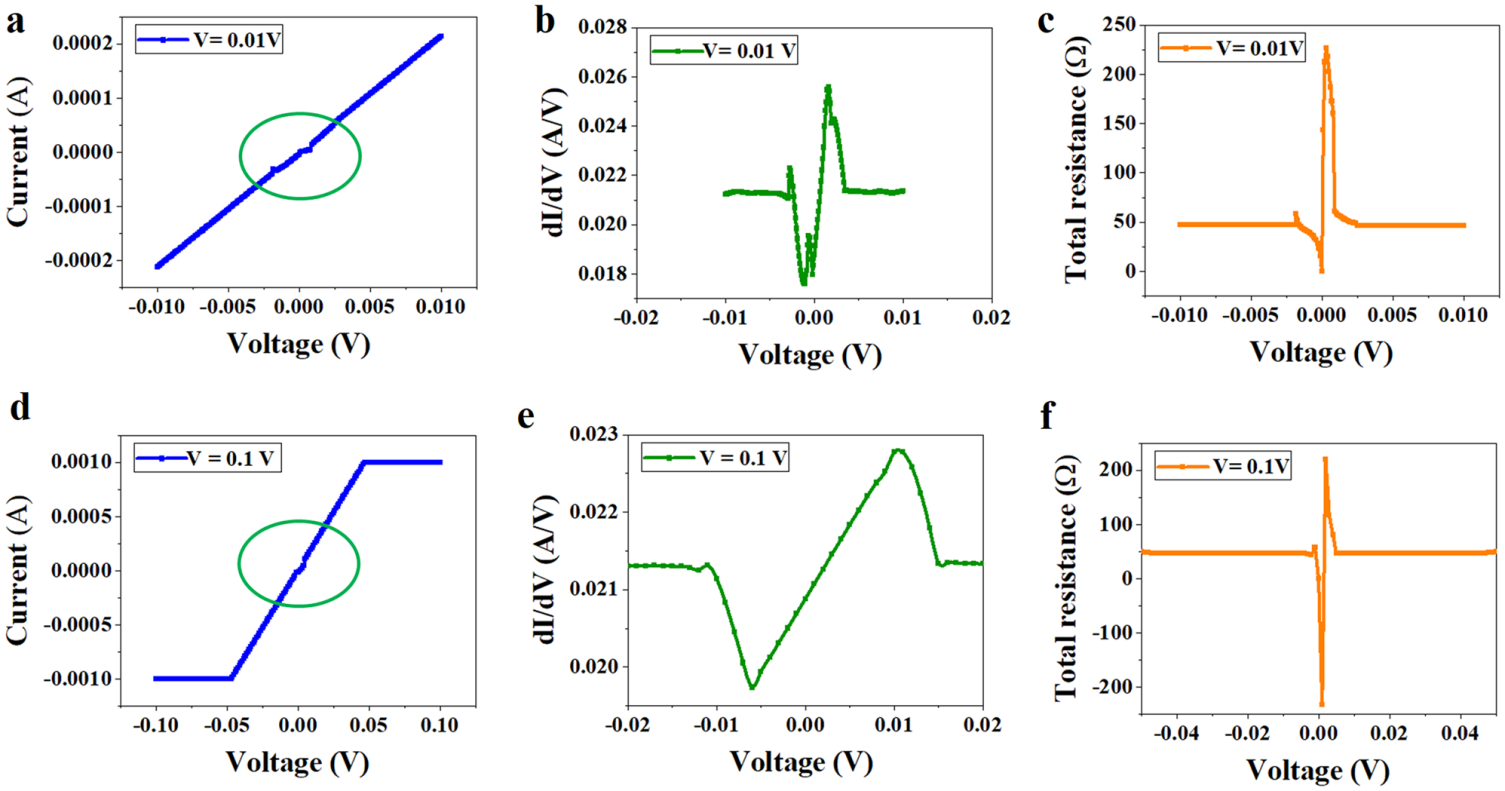
Biasing voltage-dependent VI Characteristics of the fabricated edge contact device (**a**) VI response for a maximum biasing voltage of ± 10 mV, two sharp nonlinear kinks are observed as encircled in the VI response, (**b**) corresponding first-order derivative plot, (**c**) corresponding voltage versus total resistance plot, (**d**) VI response for a maximum biasing voltage of ± 100 mV, two sharp nonlinear kinks are merged to a single one is observed as encircled in the VI response, (**e**) corresponding first-order derivative plot, and (**f**) corresponding voltage versus total resistance plot with negative resistance showing ballistic carrier transport through the edge-contact device.

We measured the edge contact devices using the Keithley 4200-SCS, a complete integrated system for the electrical measurement of samples at room temperature with a pre-amplifier connected to one of the source measure units for very sensitive measurements with low currents of the order of picoampere. As our devices show extremely low contact resistance, we characterized them with the SMU 1 (pre-amplifier connected) and the SMU2. The VI characteristics of the sample are presented in Fig. 3. It is observed that the edge contact devices show very sharp nonlinearities which are highly sensitive to the biased voltage. As shown in Fig. 3a, for a biasing voltage up to ± 10 mV, the device shows two sharp nonlinear kinks (dips) as encircled in green and evidenced from the 1st order derivative plot in Fig. 3b. Figure 3c presents the total resistance variation concerning the supplied voltage, which indicates a nonlinear resistance near zero biasing in the 2 T device. Further, as we increase the biasing voltage up to ± 100 mV, two sharp nonlinear kinks are merged into one and the degree of nonlinearity is also reduced as encircled in Fig. 3d and from the 1st order derivative plot in Fig. 3e.

As observed from Fig. 3f, the total resistance concerning voltage in the 2 T device is nonlinear as well as shows a negative near zero biasing at room temperature, which is an indication of ballistic transport through edge-contact to SL graphene. This phenomenon is similar to what has been reported by Wang et al.^13^ at low temperature (1.2 K) for FET devices based on 1D edge-contact to exfoliated graphene. This kind of nonlinear VCC and resistance variation is a sign of pure 1D edge contact in a short graphene channel length (2 μm) 2 T device. Here, the transport near the contact is dominated showing a clouding effect of charge carriers due to the electrostatic field near the metal–graphene interface for zero and low biasing voltages is evidenced, which is overcome with increasing bias voltage allowing a continuous flow of current in the graphene channel. It is believed that the electrostatic effect arises at metal–graphene contact due to doping to graphene either due to two different chemical potentials of contact material and the graphene or contamination/ oxidation of contact metal during processing leading to a potential barrier, which is quite small to be overcome with the supplied voltage. The pure-edge contacted device allows us to observe this phenomenon through nonlinear VCC and the Keithley system is highly sensitive to detect it.

**Figure 4:**
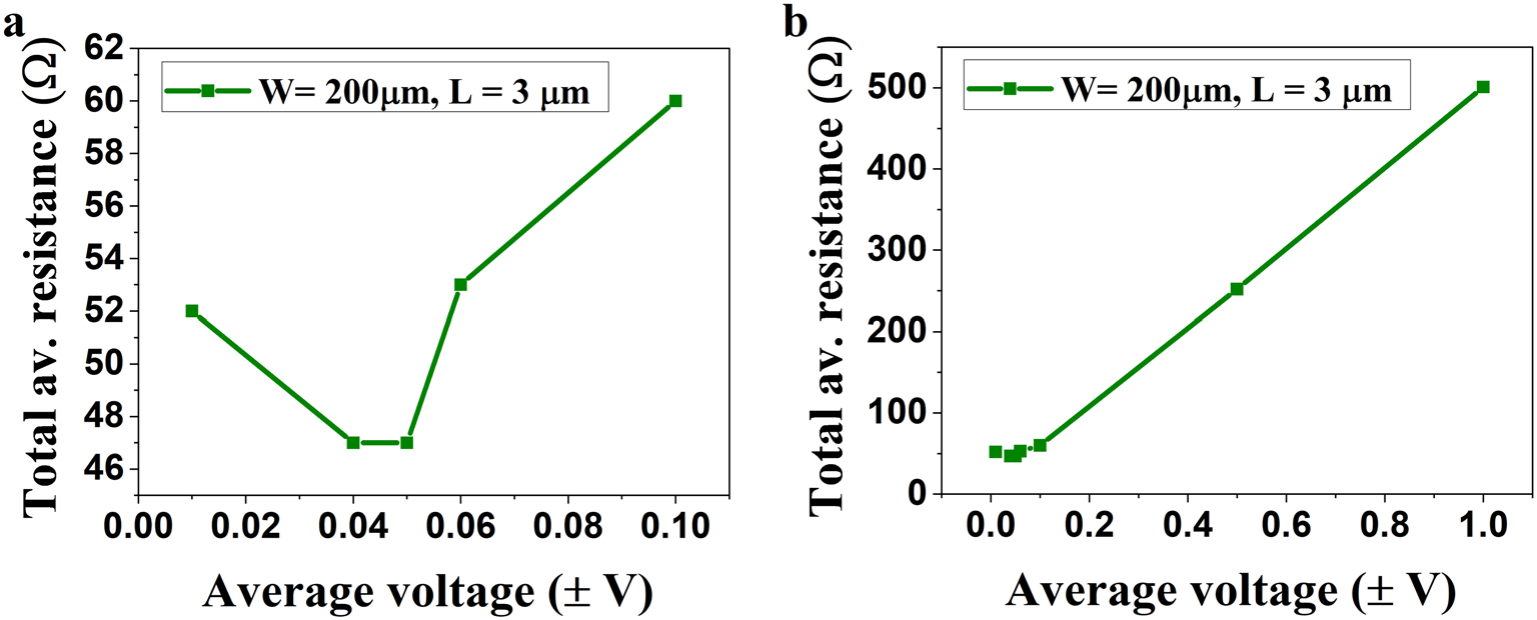
Total resistance vs. biasing voltage, (**a**) the device shows a nonlinear variation in the resistance for low biasing voltages (± 0.1 V), and (**b**) linear voltage–resistance characteristics similar to metals as the biasing voltage increases up to ± 1 V.

Figure 4 shows the variation of total average resistance (graphene + contact) in the device concerning the biasing voltage. As shown in Fig. 4a for low biasing voltages starting from ± 0.01 V, the device resistance is limited to less than 60 Ω and is a nonlinear function, whereas for higher supplied voltages (± 1 V), as shown in Fig. 4b, the device resistance increases in a similar linear fashion to that of metals. Therefore, it is concluded that the edge contact graphene device is highly sensitive to supplied voltage and shows sharp nonlinearities at very low voltages.

**Figure 5:**
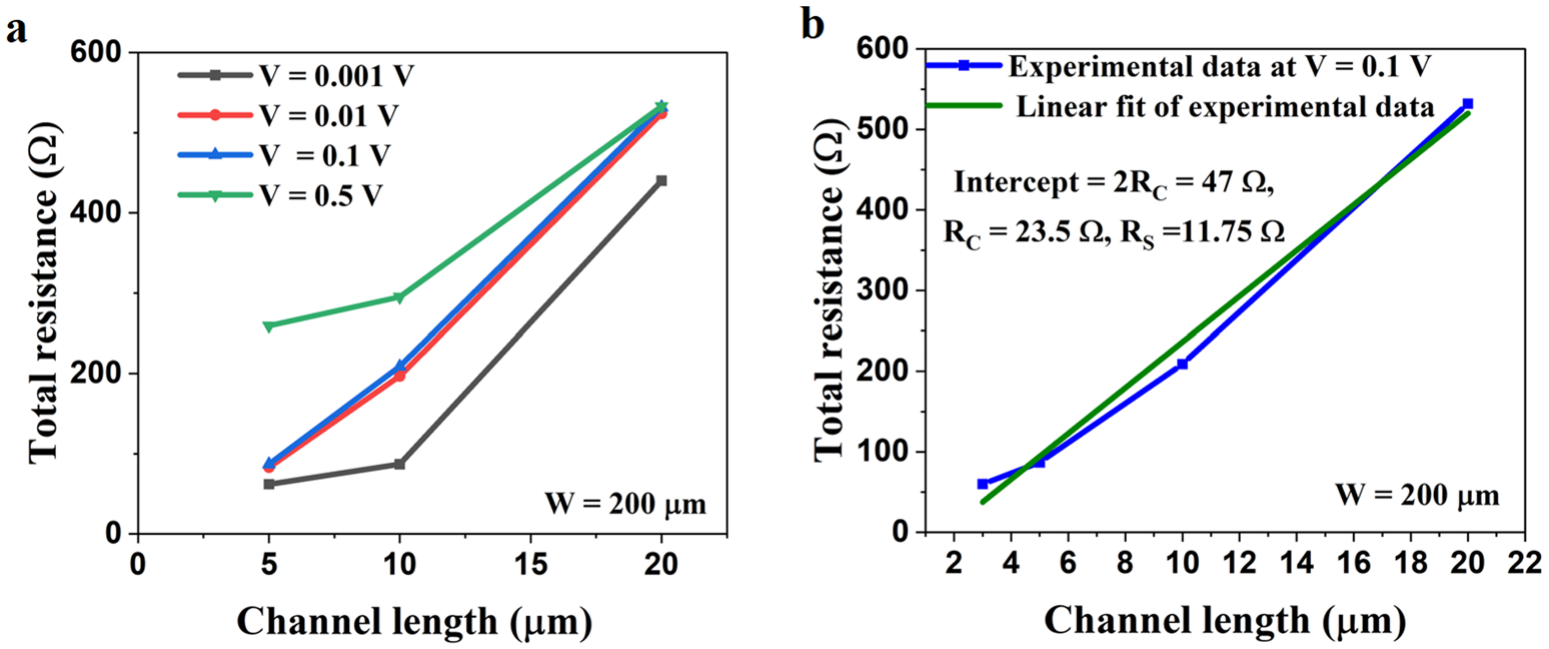
(**a**) Graphene channel length vs. total resistance for variable supplied voltages, and (**b**) linear fit of one of the repeatedly produced curves from (**a**) at a biasing voltage of 10 mV to calculate the contact and sheet resistance.

Next, we fabricated three devices with variable graphene channel lengths (5, 10, and 20 µms), integrated them into a single chip for a constant width of 200 µm, and characterized them with the Keithley measurement system at room temperature. The total resistance as a function of the biasing voltage for variable channel lengths is presented in Fig. 5a. It shows a similar behavior as studied in Fig. 3 for low and high biasing voltages. As an example, for a channel length of 5 µm, the total resistance is 62 Ω for a biasing voltage of ± 1 mV, which gradually increases to 82 Ω, 87 Ω, and 259 Ω for basing voltages of ± 10 mV, ± 100 mV, and ± 500 mV. To calculate the contact resistance and sheet resistance, we consider a biasing voltage that shows an almost linear response and performed the linear curve fitting as shown in Fig. 5b. The experimental data in Fig. 5b includes the devices with graphene channel lengths of 2, 5, 10, and 20 µm and a channel width of 200 µm. For a two-terminal device, the total resistance is given by ${R}_{total}= \frac{2{R}_{c}}{W}+\rho L(\Omega )$^9,13^. Where R_C_ is the contact resistance, $\rho$ is the resistivity, and L and W are the graphene channel length and widths. The contact resistance (R_C_) is obtained from the Y-intercept of the channel length vs. total resistance plot in Fig. 5b. The contact resistance is found to be 23.5 Ω and the sheet resistance is found to be 11.5 Ω. The two-terminal graphene device with a channel width of W = 200 µm and length of L = 2 µm shows normalized contact resistances of R_C_ × L = 47 Ω µm (minimum) and R_C_ × W = 4.7 K Ω µm (maximum).

**Figure 6:**
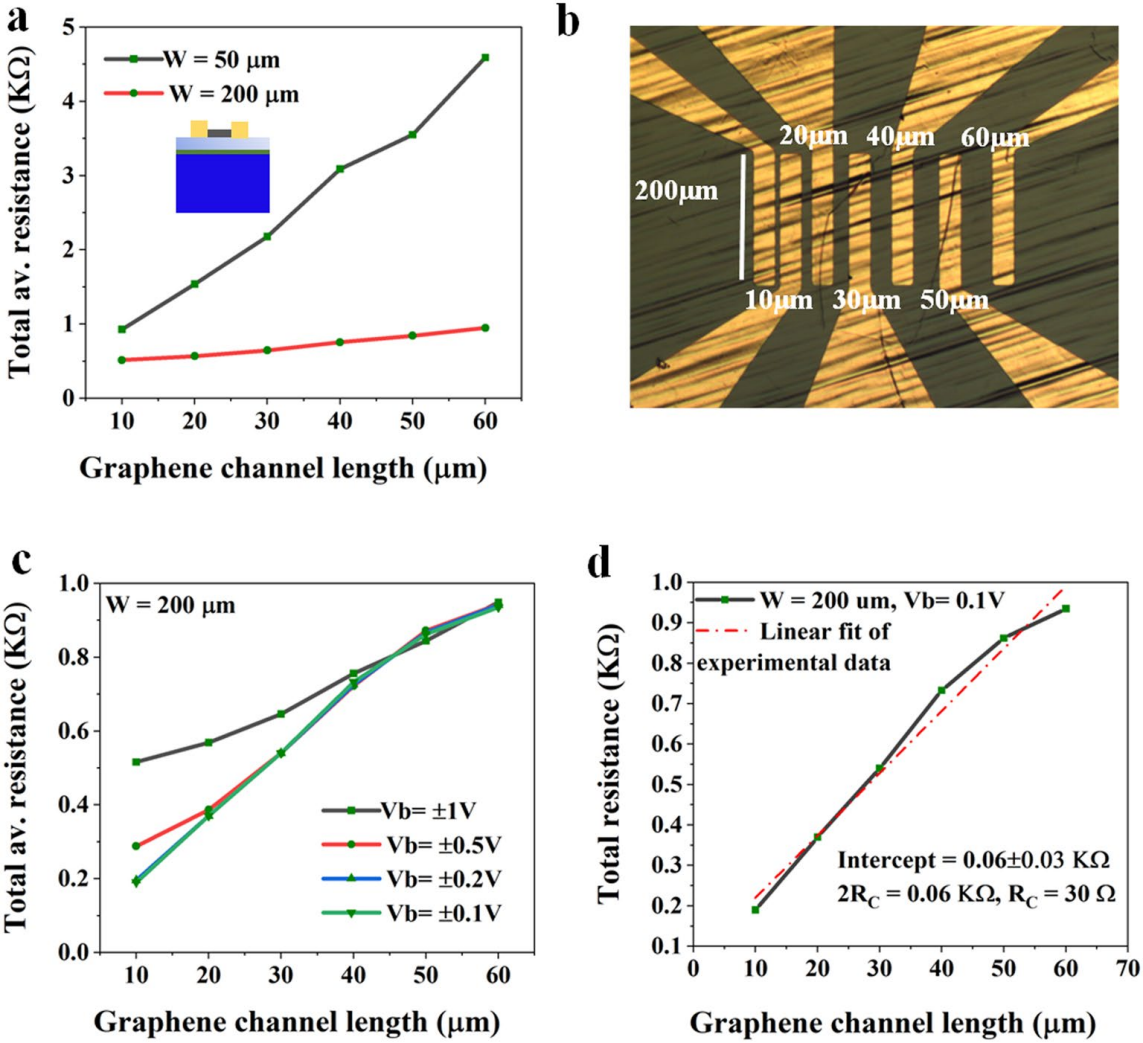
(**a**) Graphene channel length vs. total resistance of the edge-contacted TLM devices with channel widths 50 and 200 µm, (**b**) optical microscope image of the TLM device with W = 200 µm, (**c**) L vs. R_av_ plot for different biasing voltages on the edge-contacted TLM with W = 200 µm and (**d**) linear curve-fitting of the L vs. R_av_ plot of the TLM (**c**) for a biasing voltage of 0.1 V showing a contact resistance of 30 Ω.

To verify and bring clarity to the nonlinear variation of the total resistance concerning the channel lengths in the edge-contact devices, we have fabricated edge-contacted TLM devices following the same fabrication technique with Cr–Pd–Au contacts to graphene. The TLM devices with variable channel lengths (10–60 µm) and channel widths of 50 and 200 µm have carried out a similar study (Fig. 5), presented in Fig. 6. Figure 6a shows the graphene channel length versus the total average resistance plot for two sets of TLM devices with W = 50 and 200 µm. The TLM devices with W = 200 µm show comparatively low total average resistance of less than 1 KOhm than the TLM devices with W = 50 µm which shows up to 5 1 KOhm for the same set of channel lengths varying from 10 to 60 µm. Most importantly, it is observed that both sets of the edge-contacted TLM devices show a nonlinear variation in total average resistance concerning channel length. The microscope image of the edge-contacted TLM device with W = 200 µm is shown in Fig. 6b. The total resistance vs. channel length is plotted for variable supplied voltages (± 0.1–± 1 V) is presented in Fig. 6c Fig. 6d shows the linear curve-fitting of the plot (c) to obtain the contact and sheet resistance and contact resistance. The contact resistance of the TLM device is found to be 30 Ω, which gives a contact resistance of 6 KΩ µm (maximum) for W = 200 µm and 300 Ω µm (minimum) for L = 10 µm, which is similar, whereas a little higher than the contact resistance of the 2 T devices with L = 2–20 µm as the TLMs have a different channel length of 10–60 µm.

As we have contact with the graphene only at the edge, we calculate the contact resistivity as presented in^8,17^ to be the contact resistance (R_C_) × graphene channel width × width of graphene = 23.5 Ω × 200 µm × 0.0003 µm = 1.4 Ω µm^2^. It was believed that the device can show even much lower contact resistance than those reported in the literature for smaller channel widths of the devices. However, we have tried to reduce the graphene channel width to 40, 20, and 10 µm and fabricated a few devices. We observed that the contact resistance varies inversely as a function of the width of the graphene channel. This is also valid because higher channel width reduces the contact resistance through the inclusion of more resistances in parallel contributing to the contact resistance as shown in Fig. 1a, inset^26^.

**Figure 7:**
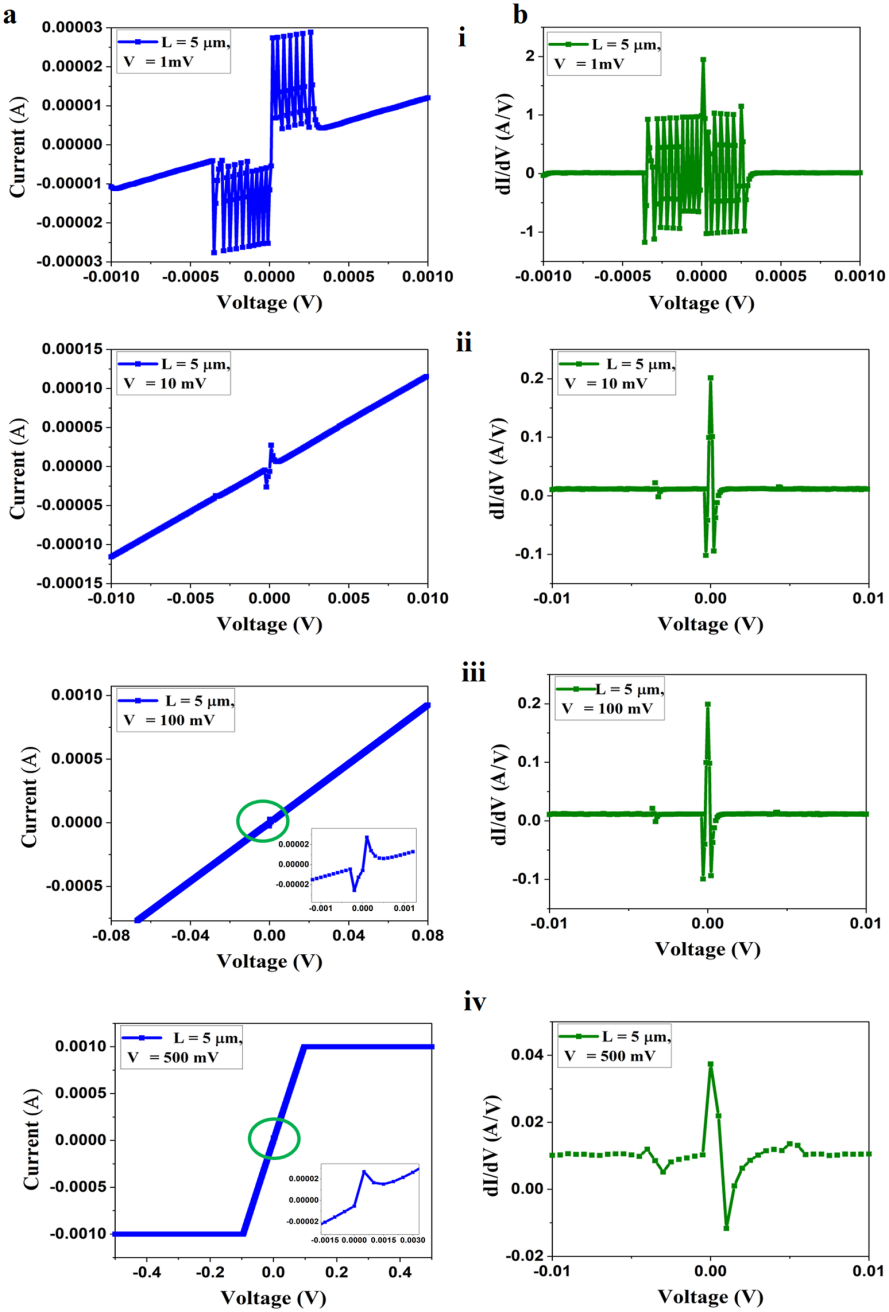
(**a**) VI Characteristics of the device for a channel length L = 5 µm with variable input voltages (i) V =  ± 1 mV, (ii) V =  ± 10 mV, (ii) V =  ± 100 mV, (iii) V =  ± 500 mV (insets show the magnified view of the green encircled region), and (**b**) (i–iv) corresponding first-order derivative plots at different bias voltages.

Further, we have also studied the VI characteristics of the edge contact devices integrated into a single chip with channel lengths 5 and 20 µm using SMU1 and SMU2 of the Keithley parameter analyzer, and the results for L = 5 are presented in Fig. 7. The VI characteristics for the device show a different voltage-sensitive sharp nonlinear response that is very prominent near zero biasing for low voltages (± 1 mV) as evidenced. by Figs. 7a-i and a-ii. This nature of VCC presents the quality of graphene integrated into our device as a very highly un-doped and single layer and pure metallic-edge-contact to the graphene atoms is achieved. When there is a pure-edge contact between the single-layer graphene and the contact material at 1D edge, the potential difference between the two materials or the barrier becomes much thinner compared to 2D contacts. This effect lowers the contact resistance and allows the flow of current even for very low voltages or near zero bias voltage as the edge-contact effect is dominated. Multiple oscillations of the current concerning charge carriers in the edge-contact region lead to the formation of an amplified standing wave which is seen as an increase in current as in Fig. 6a-i, which is a similar effect as described by Calado et al. and Poliak et al.^27,28^. The quiet mode-scanning of the Keithley system with pre-amplifier connected SMU1 is highly sensitive to detect these edge-enhanced quantum effects at the metal–graphene edge. The nonlinearity in edge-contacted devices is also a phenomenon as presented in Hemmetter et al.^17^, which is highly prominent in large–contact resistance devices.

As we increase the voltage from ± 10 to ± 100 mV, we see a similar VCC in Fig. 7a-ii and a-iii as presented in Fig. 3. Two peaks near, ± 2µA merge to a single peak at 2 µA as we further increase the supplied voltage to ± 500 mV, and the nonlinearity tend to disappear as we increase the voltage to more than 1 V as shown in Fig. 7a-iv. As the voltage increases, the carries transport to the channel region, and the transport in the channel is dominated by the transport in the contact region leading to a linear variation of current in the graphene channel. The corresponding 1st-order derivatives of the VCC are shown in Fig. 7b for clear observation of the nonlinear VCC. This kind of nonlinear effect in VCC is observed for a scanning of voltage from SMU1 to SMU2 in the Keithley system that is highly reproducible for each measurement due to the sensitivity of SMU1. A study of similar nonlinear VCC on the edge-contacted devices with 10 and 20 µm channel lengths is presented in the supplementary material (Figs. S2 and S3). However, if we change a measurement system, we are unable to see the voltage-sensitive nonlinear effects, which may be due to the high force imposed by the measurement probes on the contacts of the device.

**Figure 8:**
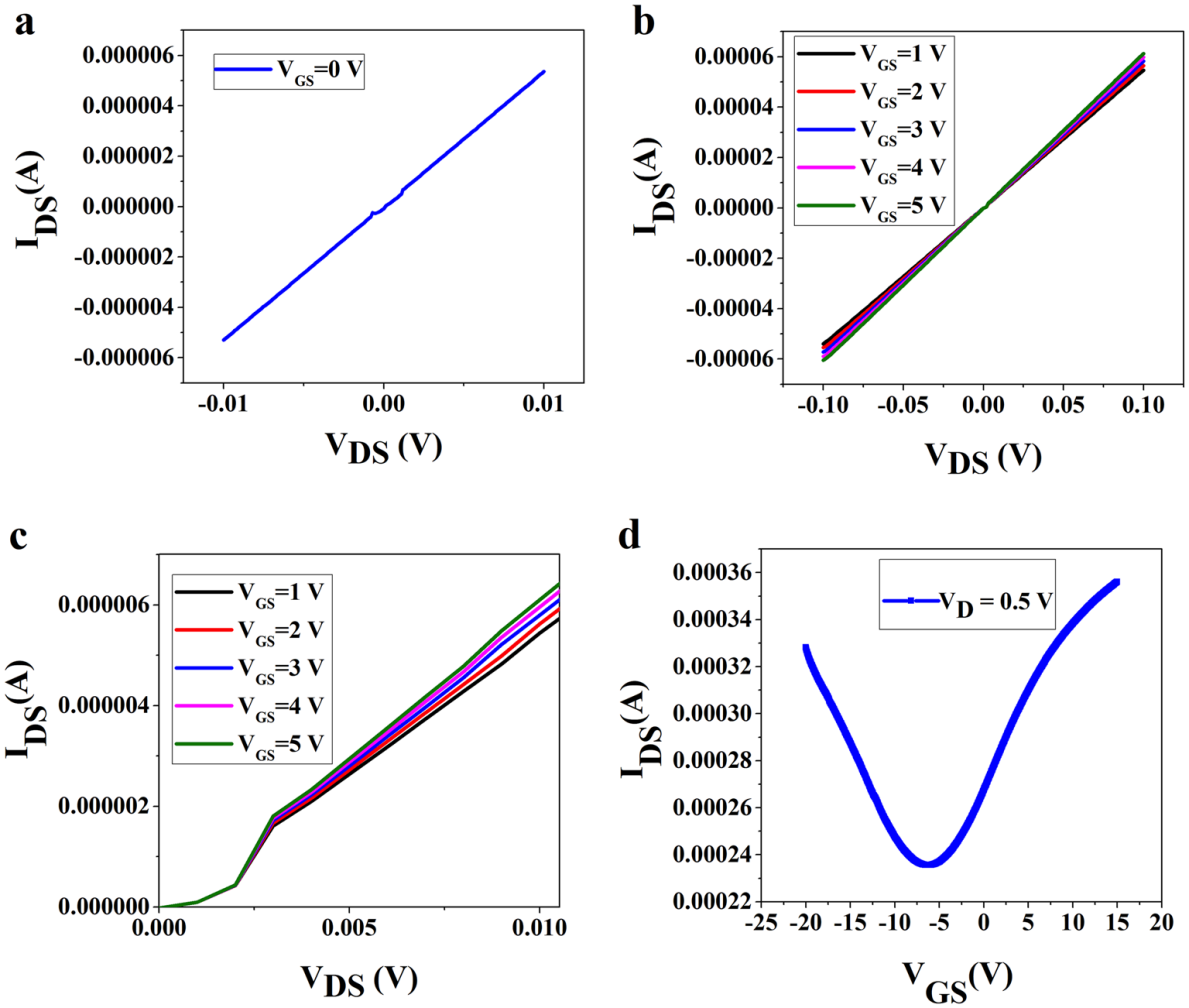
(**a**) V_DS_–I_DS_ characteristics of the edge-contacted FET with 30 µm channel length between the source-drain and 200 µm channel width at V_GS_ = 0 V, (**b**) V_DS_–I_DS_ characteristics of the edge-contacted FET at different top-gate voltages V_GS_ = 1–5 V, (**c**) magnified image of the V_DS_–I_DS_ plot showing nonlinear characteristics (**d**) the transfer characteristics (V_GS_ vs I_DS_ at V_D_ = 0.5 V) of the edge-contacted GFET showing higher electron density in the n-channel.

To further understand the origin of edge-enhanced nonlinear effects in edge-contacted devices we have fabricated edge-contacted graphene field effect transistors (GFET) and the study on current–voltage and charge transfer characteristics are presented in Fig. 8. Initially, we fabricated an edge contact device with a 30 µm channel length and 200 µm channel width following a similar technique for two-terminal devices through Cr–Pd–Au contacted to the 1D edge of graphene. Next, we have made the Ti–Au (5–80 nm) top-gate electrode insulated by 120 nm of Parylene N deposited over the edge-contacted 2 T device through CVD.

Figure 8a shows the drain-source voltage (V_DS_) vs. drain-source current (I_DS_) plot which is almost linear except for two sharp nonlinear peaks/dips near the zero biasing voltage. The origin of these two nonlinear responses in the VCC at low voltages is due to the dominant edge contact effects as discussed before. This can also be understood from the aspect of edge-contacted graphene FETs showing somewhat nonlinear-response-like semiconductors such as dark current in the absence of voltage as reported by Lee et al.^29^. Further, as we supply and increase the gate voltage from 1 to 5 V, it is observed that two peaks/dips in the VCC merge to a single nonlinear dip and shallows due to the continuous flow of current in the GFET as shown in Fig. 8b and a magnified view is shown in Fig. 8c indicating the nonlinear VI characteristics of the GFET. In large channel-length FET devices, the transport in the channel region is dominated by the transport near the edges, leading to a merging of the two edge-enhanced nonlinear dips^23^. Figure 8d presents the transfer characteristics (V_GS_ vs I_D_ at V_D_ = 0.5 V) of the edge-contacted FET with V_Dirac_ shift to negative voltage showing an n-type graphene channel of the device with Cr–Pd. Au (1–15–100 nm) contacts to graphene, where Pd–Au provides n-type doping to the graphene channel. The nonlinear GFET also shows an asymmetric transfer characteristic as evidenced by Fig. 8d. Further, the effect of biasing voltage on the top gate of our edge-contacted FET allows higher n-type doping by shifting the Dirac point to negative voltages which eventually leads to a symmetric characteristic at higher biasing voltages as presented in the supplementary material (Fig. S5). Therefore, it is concluded that our edge-contact devices are stable at room temperature, show low contact resistance, as well as nonlinear resistance vs. channel length characteristics and an indication of pure-edge contacts to single-layer graphene in terms of edge-enhanced nonlinearities in VCC at low voltages. The edge-enhanced nonlinearities in VCC are very shallow to be considered as nonlinear characteristics of the devices and can be assumed as the sensitivity of the Keithley system to capture the low-voltage-current quantum effects.

## Methods

**Figure 9:**
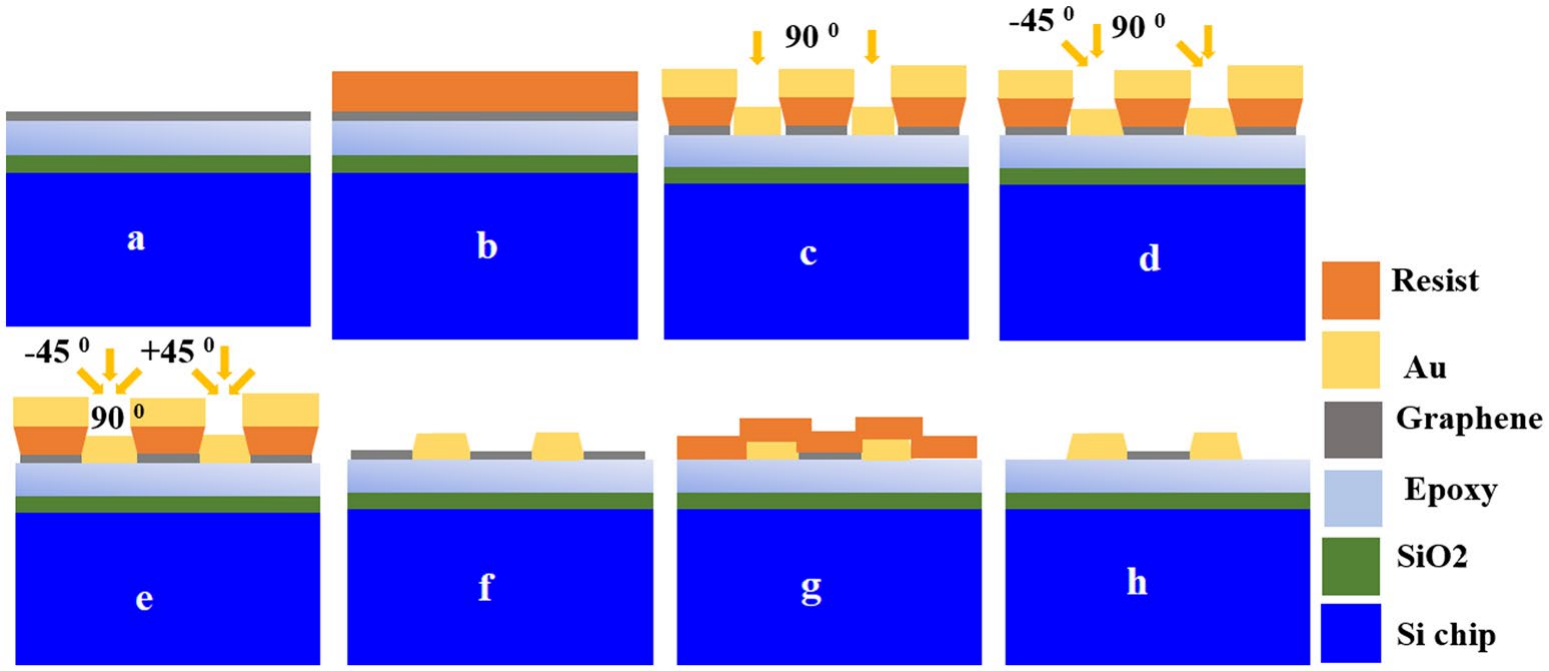
Metal–graphene (M–G) contact fabrication steps. (**a**) graphene transferred to Si–SiO_2_ using epoxy, (**b**) smart-print lithography patterning of contact electrodes using a three-layer resist coating, (**c**–**e**) O_2_ plasma etching and thermal evaporation through three different angles in a single-run using customized sample holder to get edge contact to graphene, (**f**) lift-off and developing to remove LOR layer, (**g**) single-layer resist lithography mask-protection of active graphene channel, and (**h**) lift-off, O_2_ plasma etching of graphene and resist removal to obtain the final device.

Graphene is grown on a 2-inch copper (Cu) foil using CVD as per the procedure presented in the supplementary material. The Si–SiO_2_ chips are glued to the Cu-Graphene substrate through epoxy Epotek 353ND (1:10) and dried at 60 °C for a few hours. Cu is etched in 30% HNO_3_ for a few minutes and graphene is transferred to Si–SiO_2_ as shown in Fig. 9a. A portion of graphene is transferred to polyethylene-terephthalate (PET) through direct lamination followed by copper etching for surface analysis, Raman characterization, and mobility test^30^. We have fabricated the contact to graphene in a two-step and cost-effective maskless photolithographic process using Microlight 3D smart print. Initially, we have patterned the contact pads through the maskless lithography using a three-layer resist coating including S1805, LOR 3A, and S1813 as shown in Fig. 9b to obtain an undercut, which allows an easy lift-off. Before the metal evaporation, we carried oxygen plasma etching of graphene below the contact pads for 40 s at 50 W with a flow of 15 sccm O_2_ under a pressure of 250 mBar. However, the undercut prohibits contact metal to touch the graphene edge under normal incidence thermal evaporation. After many failures to achieve edge contact with graphene through normal incidence evaporation on the plasma etched metal contacts, we deal with this challenge through a customized sample holder which allows us to rotate the sample plane up to ± 45°. Further, we have also chosen Cr–Pd–Au (1/7.5/50 nm) as the contact metal to graphene, which is proved to be a suitable choice of contact material for graphene to achieve high mobility^31^. Cr–Pd–Au is deposited on the sample from three different angles (90°, 45°, and − 45°) to reach the edge of the graphene from all directions as shown in Fig. 9c–e. Lift-off is carried out to obtain the 2 T device as shown in Fig. 9f. We carried out the next step lithography as shown in Fig. 9g to protect graphene only in the active region and etched it everywhere, using O_2_ plasma etching. Now, the device as shown in Fig. 9h with Cr–Pd–Au edge contact to graphene is ready for measurements. Raman characterizations of the PET/graphene samples are carried out using a Raman spectrometer, Horiba Scientific with a 638 nm Laser with 100 × objective for 10 s using 1200 gratings per mm and a 300 µm slit. Low-resolution SEM images of the graphene on PET are taken with a Zeiss Supra 60 VP-EDS to reveal the surface morphology of the transferred graphene. We use a customized Hall measurement system with four probes towards measuring the mobility, charge carrier density, and sheet resistance of the graphene transferred to PET. VI characteristics of all the fabricated edge-contacted devices are performed at room temperature using a Keithley 4200-SCS parameter analyzer.

## Conclusion

In the presented study, we could achieve pure metal contact to the 1D edge of single-layer 2D graphene through a cost-effective and simple fabrication approach and have studied the voltage-current characteristics of the integrated graphene to two-terminal (2 T), TLM and FET devices. The Cr–Pd–Au edge contacted 2 T device shows a contact resistance of 23.5 Ω between the two terminals, a nonlinear total resistance variation concerning voltage and channel lengths, a nonlinear voltage-current relationship that is highly sensitive to the input voltages, and shows some signs of charge clouding and quantum transport effects through atomic edge-of graphene. Nonlinear VCC in graphene devices is applicable in frequency mixing and switching. This study presents a robust approach toward realizing geometry-dependent graphene-integrated edge contact devices with low contact resistance for future high-speed and low-power graphene-integrated electronic devices and circuits.

## Supplementary Information

Supplementary Information 1.

Supplementary Table S1.

## Data Availability

The data shall be available upon request to the corresponding author.
