# Supplementary material for: Pure edge-contact devices on single-layer-CVD-graphene integrated into a single chip
Source: Sci Rep. 2023 Jun 30;13:10588. doi: 10.1038/s41598-023-37487-1 (PMC10313717; doi:10.1038/s41598-023-37487-1)
Supplement: Supplementary file 1 — Supplementary Information 1. [file 41598_2023_37487_MOESM1_ESM.docx]

Pure edge-contact devices on single-layer-CVD-graphene integrated into a single chip

1. **CVD Graphene growth/ deposition technique**

A 2-inch Copper (Cu) foil is cleaned in acetone through sonication for 10 minutes followed by 15 minutes in 50% acetic acid. Graphene is grown on Cu through a 2-step chemical vapor deposition (CVD) technique with CVD Aixtron Blackmagic at 70 mBar pressure and a temperature of the thermocouple at 880 ˚C, which corresponds to 1037 ˚ C of the ambient. Cu is annealed inside the CVD chamber at 1037 ˚ C for half an hour. In the first step, 30 sccm of 5% CH4 in argon and 60 sccm of $H_{2}$ are allowed for 3 minutes for the diffusion and island formation followed by the growth of single-layer graphene for 6 minutes at 120 sccm of 5% ${CH}_{4}$ and 60 sccm of $H_{2}$.

1. **2D Raman map of the single-layer graphene**

A 2D Raman map of the grown graphene transferred to PET is shown in Figure S 1.
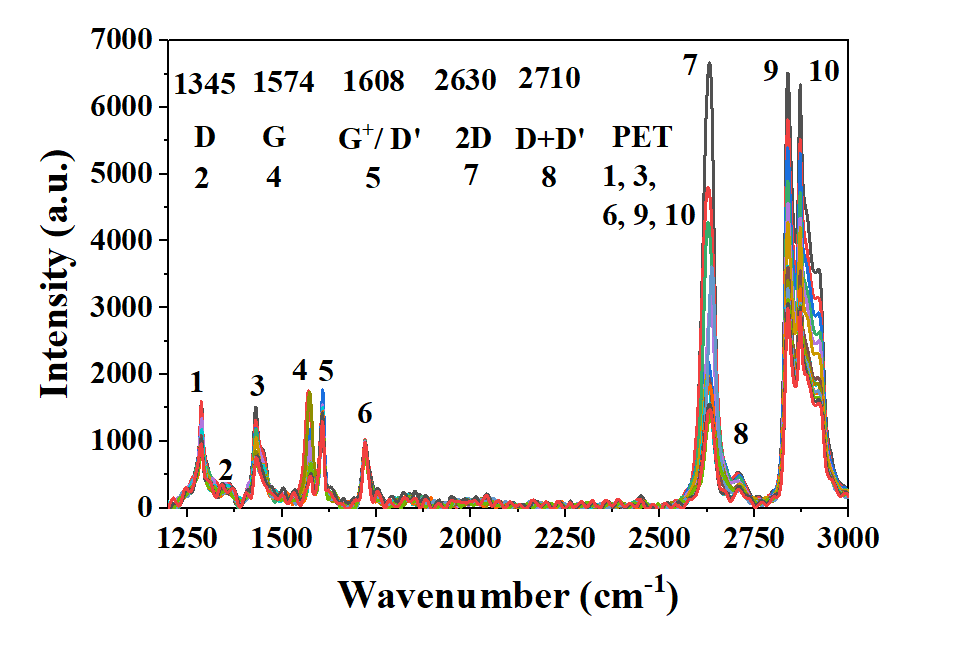


Figure S 1. 2D Raman map of the sample over an area of 10 µm^2^ showing signatures of high-quality single-layer graphene with negligible D Pea, high-intensity 2D Peak (2630 cm^-1^) and very low-intensity G peak (1574 cm^-1^) with an additional G^+^ blue shifted G peak (1608 cm^-1^) or a D' peak in the presence of PET. The additional peaks (1,3, 6, 9, and 10) are due to the PET substrate.

1. **Comparison of contact resistance in edge-contacted devices**

Table S 1 presents a comparison study of the contact resistances in edge-contacted devices available in the literature. The edge-contacted devices based on exfoliated graphene shows least contact resistance as presented by L. Wang et.al [13]. However, our 1D edge contacted devices based on single layer CVD graphene shows lowed contact resistance compared to a similar study presented by A Hemmetter et.al. [17].

| Paper | Type of contact | Contact resistance |
| --- | --- | --- |
| A. Hemmetter et.al. [17] | 1D edge to CVD graphene. | 74 K Ω, for W = 50 µm  3.7 M Ω. µm |
| L. Wang et.al [13] | 1D edge contact to exfoliated graphene sandwiched by HBN. | 150 Ω. µm for W = 2 µm |
| D. W. Yue et.al [9] | Partial edge contact with plasma treatment on CVD graphene. | 1- 3 K Ω. µm |
| W. S. Leong et.al. [16] | Edge-treated (metal catalyzed etching of Ni contact in Hydrogen) exfoliated Sl layer graphene.  Untreated exfoliated SL layer graphene. | 100 Ω. µm for W = 2 µm  600 Ω. µm for W = 2 µm |
| K. Nagashio et.al. [15] | Edge contact to exfoliated graphene. | 1 K Ω. µm for W = 2 µm |
| S. Behera et.al. | 1D edge-contact to CVD graphene. | **23. 5 Ω or 4.7 K Ω. µm**  **for W = 200µm** |

Table S 1. Comparison of contact resistance in edge-contact devices.

1. **VI Characteristics of devices with variable channel lengths (5 and 20 µm) showing similar voltage-current characteristics (VCC) at variable bias voltages.**

The VCC of the two devices with channel widths of 200 µm and lengths of 5 and 20 µm is shown in Figure S 2. Both of the devices have a similar nonlinear VCC at 100 mV supplied voltage.


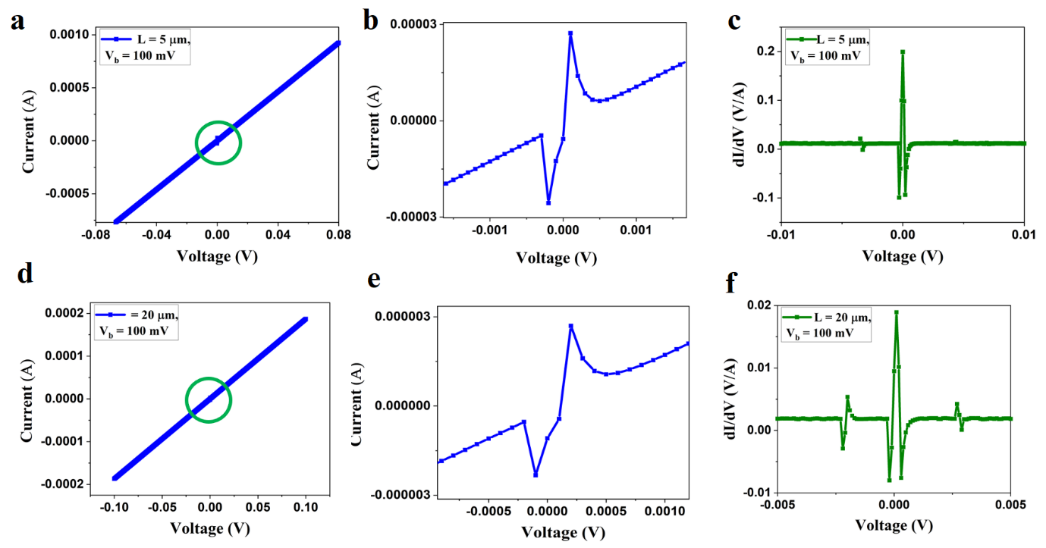


Figure S 2. VI Characteristics of the fabricated edge contact devices with channel widths of 200 µm and lengths of 5 and 20 µm for a supplied voltage of ± 100mV.

1. **VI Characteristics of the device with 10 µm channel length showing a different nonlinear VCC at variable bias voltages**

The VCC of the device with a channel width of 200 µm and length of 10 µm is shown in Figure S 3. This device shows a little different nonlinear VCC concerning the supplied voltage. However, at high voltages, it shows similar characteristics to those of 5 and 20 µm channel length devices in terms of reduced nonlinearities and tending toward linearity in VCC. Therefore, from all these studies, we confirm voltage-sensitive nonlinear voltage-current characteristics reflecting the electrostatic effect and nature of electron transport from metal contacts through 1D atomic edges of single layer-graphene.


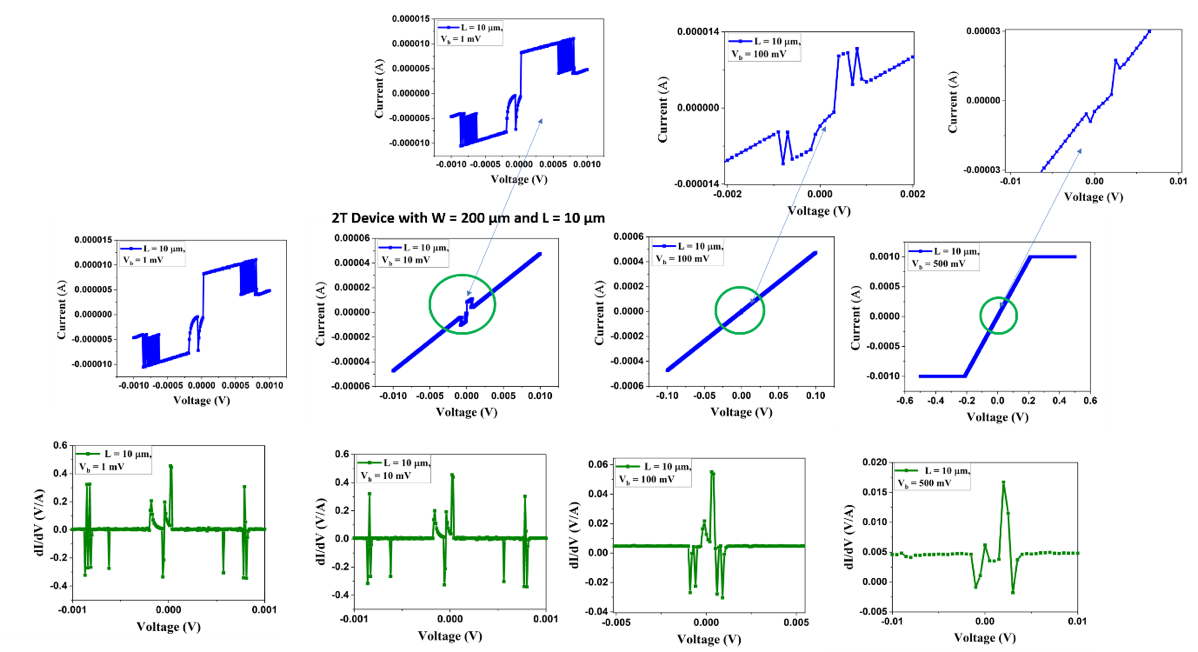


Figure S 3. VI Characteristics of the fabricated edge contact device with channel widths of 200 µm and lengths of 10 µm for a variable supply voltage of 1- ± 500 mV

1. **Hall measurement on the mobility of the graphene transferred to PET**

Figure S 4 shows the signatures of high mobility in our transfer of graphene to PET.


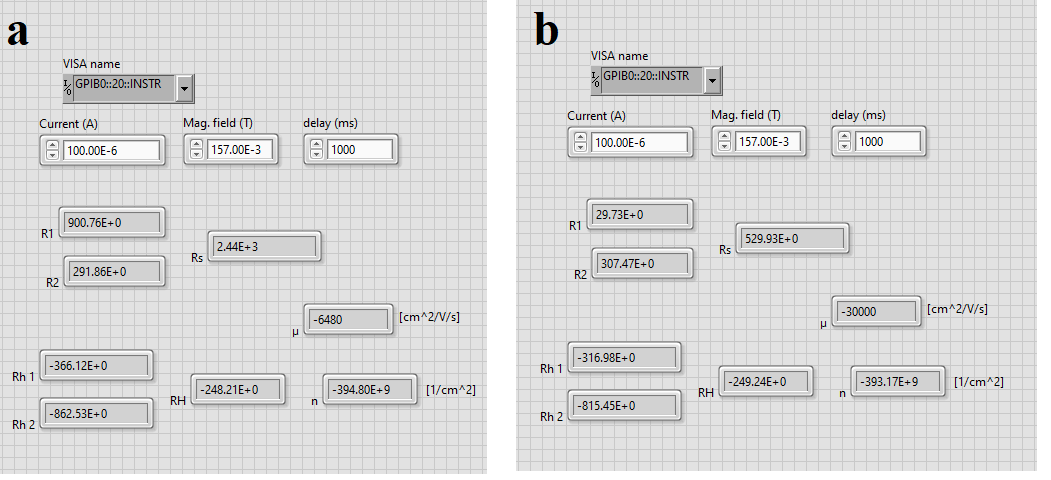


Figure S 4. The graphene transferred to PET showed high and stable mobility (a) after degas at 60 degrees for a few hours and (b) immediate measurement after degas showing very high mobility up to 30000 cm^2/^V/s.

**7. Biasing voltage-dependent electronic modulation in GFET device**

We have also studied the effect of biasing voltage to the top gate of our edge-contacted GFET device as presented in Figure S 5. The GFET shows n-type doping for a V_GS_ of -15-10 V with the Dirac point at -5 V with asymmetric characteristics which gradually shift to -8 V for higher biasing voltage (-20-15 V). Further increase of gate voltage from -35 to 15 V shifts the Dirac point to negative voltages >-10V and eventually leads to a symmetric characteristic. This is a similar effect as described by Peng-et. al through interface trap engineering to electronic property modulation in Graphene [25].


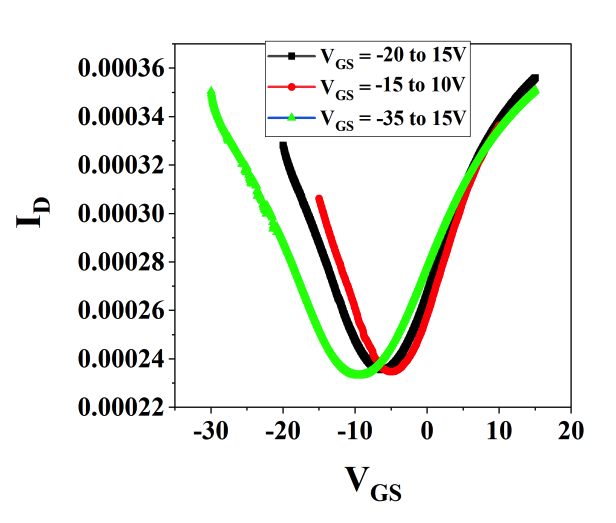


Figure S 5. Biasing voltage-dependent electronic modulation in graphene field effect transistors (GFET).
