# Supplementary material for: Pure edge-contact devices on single-layer-CVD-graphene integrated into a single chip
Source: Sci Rep. 2023 Jun 30;13:10588. doi: 10.1038/s41598-023-37487-1 (PMC10313717; doi:10.1038/s41598-023-37487-1)
Supplement: Supplementary file 2 — Supplementary Table S1. [file 41598_2023_37487_MOESM2_ESM.docx]

**Table S 1. Comparison of contact resistance in edge-contacted devices**

| Paper | Type of contact | Contact resistance |
| --- | --- | --- |
| A. Hemmetter et.al. [17] | 1D edge to CVD graphene. | 74 K Ω, for W = 50 µm  3.7 M Ω. µm |
| L. Wang et.al [13] | 1D edge contact to exfoliated graphene sandwiched by HBN. | 150 Ω. µm for W = 2 µm |
| D. W. Yue et.al [9] | Partial edge contact with plasma treatment on CVD graphene. | 1- 3 K Ω. µm |
| W. S. Leong et.al. [16] | Edge-treated (metal catalyzed etching of Ni contact in Hydrogen) exfoliated Sl layer graphene.  Untreated exfoliated SL layer graphene. | 100 Ω. µm for W = 2 µm  600 Ω. µm for W = 2 µm |
| K. Nagashio et.al. [15] | Edge contact to exfoliated graphene. | 1 K Ω. µm for W = 2 µm |
| S. Behera et.al. | 1D edge-contact to CVD graphene. | **23. 5 Ω or 4.7 K Ω. µm**  **for W = 200µm** |
